# Polymerization potential of a bacterial CotA-laccase for β-naphthol: enzyme structure and comprehensive polymer characterization

**DOI:** 10.3389/fmicb.2024.1501112

**Published:** 2024-11-21

**Authors:** Marina Refaat, Marwa T. ElRakaiby, Mustapha El Hariri El Nokab, Julien Es Sayed, Ahmed Elshewy, Khaled O. Sebakhy, Nayera Moneib, Tuo Wang, Thomas J. Smith, Mohamed H. Habib

**Affiliations:** ^1^Department of Microbiology and Immunology, Faculty of Pharmacy, Cairo University, Cairo, Egypt; ^2^Department of Chemistry, Michigan State University, East Lansing, MI, United States; ^3^Zernike Institute for Advanced Materials (ZIAM), University of Groningen, Groningen, Netherlands; ^4^Department of Pharmaceutical Organic Chemistry, Faculty of Pharmacy, Cairo University, Cairo, Egypt; ^5^Department of Natural and Applied Sciences, College of Arts and Sciences, The American University of Iraq-Baghdad (AUIB), Baghdad, Iraq; ^6^Department of Materials, Textiles and Chemical Engineering, Centre for Polymer and Material Technologies (CPMT), Ghent University, Ghent, Belgium; ^7^Department of Materials, Textiles and Chemical Engineering, Laboratory for Chemical Technology (LCT), Ghent University, Ghent, Belgium; ^8^Department of Biochemistry and Molecular Biology, University of Texas Medical Branch (UTMB) at Galveston, Galveston, TX, United States; ^9^Department of Internal Medicine, John Sealy School of Medicine, University of Texas Medical Branch at Galveston (UTMB), Galveston, TX, United States

**Keywords:** poly-β-naphthol, enzymatic polymerization, one-gram conversion, optimization, expression, crystal structure

## Abstract

**Introduction:**

Laccases are blue-multicopper containing enzymes that are known to play a role in the bioconversion of recalcitrant compounds. Their role in free radical polymerization of aromatic compounds for their valorization remains underexplored. In this study, we used a pBAD plasmid containing a previously characterized CotA laccase gene (abbreviated as *Bli*-Lacc) from *Bacillus licheniformis* strain ATCC 9945a to express this enzyme and explore its biotransformation/polymerization potential on β-naphthol.

**Methods:**

The protein was expressed from TOP10 cells of *Escherichia coli* after successful transformation of the plasmid. Immobilized metal affinity chromatography (IMAC) was used to generate pure protein. The biocatalytic polymerization reaction was optimized based on temperature, pH and starting enzyme concentration. ^1^H and ^13^C solution nuclear magnetic resonance (NMR), Fourier transform infrared spectroscopy (FTIR), and solid-state NMR (ssNMR) were used to characterize the formed polymer. A one-gram conversion reaction was done to explore applicability of the reaction in a pilot-scale.

**Results:**

The polymerization reaction generated a brown precipitate, and its chemical structure was confirmed using ^1^H and ^13^C NMR and FTIR. SsNMR revealed the presence of two different orientational hydroxyl functional groups in the polymer in addition to the presence of a very small amount of ether linkages (< 2%). This analysis elucidated that polymerization occurred mainly on the carbons of the aromatic rings, rather than on the carbons attached to the hydroxyl groups, resulting in a condensed ring or polynuclear aromatic structure. The reaction was optimized, and the highest yield was attained under conditions of 37°C, pH 10 and a starting enzyme concentration of 440 nM in 50 mM phosphate buffer. A one-gram conversion yielded 216 mg of polymer as dry mass. The crystal structure of the enzyme was solved at 2.7 Å resolution using X-ray crystallography and presented with a hexagonal space group. The final structure was deposited in the Protein Databank (PDB) with an ID−9BD5.

**Discussion:**

This article provides a green/enzymatic pathway for the remediation of phenolics and their valorization into potential useful polymeric materials. The comprehensive analysis of the formed polymer provides insight into its structure and functional moieties present. Based on the yield of the one-gram conversion, this synthetic method proves useful for a pilot-scale production level and opens opportunities to invest in using this polymer for industrial/environmental applications.

## 1 Introduction

Over recent years, interest has been growing for the use of recombinant enzymes for biotransformation reactions such as the dechlorination of polychlorinated biphenyls (PCBs) by converting them into less harmful compounds, or the synthesis of pharmaceutical intermediates (Trono, 2019). This phenomenon was attributed mainly to the selective and somewhat promiscuous nature of recombinant enzymes to perform new-to-nature reactions not known to occur chemically such as asymmetric reductions, C–C bond formations, and the oxidative cleavage of complex organic molecules (Leveson-Gower et al., 2019). One significant class of enzymes still considered under-explored but with a promising potential in bioremediation is the laccase family (Uyama and Kobayashi, 2003; Güreşir et al., 2005; Xu et al., 2005; Grmasha et al., 2024; Liu et al., 2024; Magalhães et al., 2024; Serbent et al., 2024; Zheng et al., 2024). Beyond laccases, other well-known families of enzymes, such as peroxidases and dehydrogenases, are also gaining attention for their roles in environmental applications and the degradation of pollutants (Alsadik et al., 2021).

Laccases, also classified as benzenediol/oxygen oxidoreductases with an Enzyme Commission (EC) number of 1.10.3.2, are multi-copper oxidases having 4 copper atoms arranged with oxygen at its center (Enguita et al., 2003; Brissos et al., 2022). The 4 copper atoms are termed Cu T1 (where the reducing substrate binds) and trinuclear copper cluster T2/T3 (Gianfreda et al., 1999). They only need molecular oxygen to operate, hence their green nature and large interest in industry. Laccases have been shown to catalyze the oxidation of phenols, polyphenols, and non-phenolic aromatic compounds through a free radical mechanism, where the enzyme facilitates the transfer of electrons from the substrates to molecular oxygen, resulting in the formation of water and oxidized products. This process typically involves the generation of free radicals that can further react with other substrates, allowing for a broad range of oxidative transformations (Gianfreda et al., 1999; de Gonzalo et al., 2016; Mahuri et al., 2023). Since it can act upon a wide variety of phenolic and non-phenolic substrates, laccase has potential applications in the food, pharmaceutical and environmental industries (Mayolo-Deloisa et al., 2020).

β-naphthol, is a phenolic compound considered as an environmental priority pollutant. β-naphthol is widely found in wastewater generated from industrial processes involving petrochemicals, oils, plastics, pharmaceuticals, and synthetic resins (Cetinkaya and Ozdemir, 2018). Various approaches, including membrane technology, extraction, advanced oxidation processes, ion exchange, and adsorption have been employed for removing these toxic compounds (Bashir et al., 2013). However, conventional methods of pollutant removal often fail to reduce pollutants to acceptable levels, whereas biological methods offer more efficient, environmentally friendly and biocompatible alternatives (Mousavi et al., 2021). Enzyme-catalyzed oxidative polymerizations (EOPs) are particularly crucial in bioremediation (Uyama and Kobayashi, 2002), and few studies have explored laccases for polymerizing phenolic compounds (Xu et al., 1995; Wang et al., 1997). For example, Aktaş and colleagues specifically focused on using laccases for the polymerization of the toxic α-naphthol (Aktaş et al., 2000), the removal of which was beneficial for wastewater treatment as well as for producing poly-α-naphthol (Aktaş et al., 2001; Arregui et al., 2019). Crystal structures for laccases from various sources have been determined over recent years but only a few were CotA laccases (Enguita et al., 2003). In this work, we present the crystal structure of a CotA-laccase enzyme derived from *Bacillus licheniformis* ATCC 9945a (referred to in this paper as *Bli-*Lacc) adding to the growing database of CotA-laccases. We further explored the effect of *Bli*-Lacc on the polymerization of β-naphthol.

## 2 Materials and methods

The pBAD-*Bli*-Lacc plasmid used in this study was provided by GECCO-Biotech (Groningen, The Netherlands). *E. coli* TOP10 (Thermofisher Scientific, Waltham, MA, USA), and its phage-resistant form NEB-10β (New England Biolabs, Ipswich, MA, USA), were used for preparation of chemically competent cells needed for transformation and later expression of the protein.

### 2.1 Plasmid transformation, protein expression, and purification

#### 2.1.1 Transformation and expression of pBAD- *Bli*-Lacc

For crystallization experiments, laccase was originally isolated from *B. licheniformis* ATCC 9945a and cloned into the pBAD expression vector (Lončar et al., 2016). The plasmid was transformed into chemically competent NEB 10-β *E. coli* cells. The transformed cells were streaked on a Luria Bertani (LB) agar (Sigma Aldrich, St Louis, MO, USA) plate supplemented with 100 μg mL^−1^ ampicillin (Sigma Aldrich). Colonies were selected and grown overnight at 37°C in LB broth (Sigma Aldrich) containing 100 μg mL^−1^ ampicillin. This inoculum was added to four liters of terrific broth (TB)—prepared in-house using yeast extract, tryptone, glycerol and mono- and dipotassium phosphate salts (Sigma Aldrich; Sambrook and Russell, 2001)—containing ampicillin in a 1:100 ratio. Cells were grown at 37°C until the optical density (OD) reached 0.6 at 600 nm measured using an Implen OD600 photometer (Munich, Germany). The flasks were then cooled down on ice before adding 0.02% (w/v) arabinose (Sigma Aldrich). Expression was performed overnight at 17°C/180 rpm using a New Brunswick Innova 44 shaker (Eppendorf, Hamburg, Germany).

For polymerization experiments, plasmid transformation, preculture and expression cultures were prepared as for crystallization experiments with some modifications (Lončar et al., 2016). Chemically competent *E. coli* TOP10 cells were used instead of NEB 10-β cells. The culture flask (containing 200 mL TB medium) upon reaching an OD_600nm_ of 0.6 was transferred to a WiseCube, WIS-30R shaker (witeg Labortechnik GmbH, Wertheim, Germany) operating at 17°C and 180 rpm for 48 h.

#### 2.1.2 Harvesting and purifying the expressed laccase

For crystallization experiments, cells were harvested by centrifugation at 6,000 rpm for 20 min using a Sorvall LYNX 4000 Superspeed Centrifuge (Thermofisher Scientific). The pellets were resuspended in 25 mM potassium phosphate, pH 7.5, that contained 500 mM NaCl, 1 mM CuCl_2_, 1 mg mL^−1^ lysozyme, 1 mM phenylmethylsulfonylfluoride dissolved in 100 μL dimethyl sulfoxide (DMSO; Sigma Aldrich), and the Halt™ protease inhibitor cocktail (ThermoFisher Scientific). The suspension was stirred at 4°C for 1 h using a C-MAG MS 4 magnetic stirrer (IKA, Willmington, NC, USA) and pulse sonicated (Hielscher Ultrasonics UP50H probe sonicator) for 30 min while maintaining the temperature at 4–10°C. The suspension was then incubated at 42°C for 1 h in a water bath and debris were removed by centrifugation at 10,000 *g* for 20 min. Laccase was purified from the supernatant using gravity-flow 5 mL columns of Ni-NTA cOmplete resin (Roche, Basel, Switzerland). The loading and wash buffer was composed of 25 mM potassium phosphate, pH 7.5, with 500 mM NaCl. Protein was eluted with the same buffer with the addition of 100 mM imidazole (Sigma Aldrich). Fractions containing laccase were pooled and dialyzed overnight against 20 mM Tris (Sigma Aldrich), pH 7.5 containing 5 mM EDTA (Sigma Aldrich).

For polymerization experiments, the laccase was purified in a slightly modified manner. After 48 h, the laccase expression culture was harvested by centrifugation at 4,000 rpm for 20 min at 4°C (Centurion Scientific, West Sussex, UK). The pellet was disrupted by sonication in potassium phosphate 50 mM, pH 7.8, containing 300 mM NaCl, 2 mM CuCl_2_ and 0.5% Triton X-100 (Sigma Aldrich) then incubated at 50°C for 1 h. The cell-free suspension was centrifuged at 6,000 rpm for 1 h. The cell free extract was added to a prepacked Cytiva 1 mL Ni-NTA cartridge (Marloborough, MA, USA) after equilibrating the resin using 5 column volumes of potassium phosphate 50 mM, pH 8.0 containing 500 mM NaCl and 5% glycerol (v/v; Sigma Aldrich). The column was washed using 10 column volumes of 50 mM potassium phosphate, pH 8.0, containing 500 mM NaCl, 5% glycerol, and 10 mM imidazole. The laccase was then eluted using 50 mM potassium phosphate, pH 8.0 containing 500 mM NaCl, 5% glycerol, and 500 mM imidazole. The protein was desalted using a G25 Desalting Purification Cartridge (Biocomma, Guangdong, China) using 50 mM potassium phosphate pH 7.5 with 150 mM NaCl, and 10% glycerol (v/v). Laccase purity was determined via SDS-PAGE (Supplementary Figure 1). Both purification methods were implemented using the purification protocol executed by Lončar et al. (2016).

#### 2.1.3 Anion-exchange column purification for crystallization experiments

The laccase was subsequently purified using an anion exchange column (MonoQ) attached to a Cytiva AKTA-FPLC (Marloborough, MA, USA) system using 50 mM Tris, pH 7.5 ± 1 M NaCl. The proteins were eluted from the column using a 0–30% linear gradient over 60 milliliters and collected as 1 mL fractions. Activity of the various peaks were tested using 100 μM syringaldazine (Sigma Aldrich) as a substrate in 100 mM Tris pH 7.5 buffer. Purity was determined using sodium dodecyl sulfate polyacrylamide gel electrophoresis (SDS-PAGE). The purest and most active samples were pooled and dialyzed against 20 mM Tris, pH 7.5 containing 200 mM NaCl overnight at 4°C. Precipitated protein and CuCl_2_ were removed by centrifugation. The remaining soluble protein was concentrated using Amicon^®^ Ultra Centrifugal Filter (Sigma Aldrich) Centricon concentrators with a cutoff size of 30 kDa. The protein concentration was estimated using absorption at 280 nm (1 mg mL^−1^ = 1 OD) using a Nanodrop 2000 (Thermofisher Scientific).

### 2.2 Polymerization of β-naphthol

The ability of the expressed laccase to polymerize β-naphthol **(1)** (ADWIC, Abu Zaabal, Egypt) was tested by incubating 440 nM of enzyme with 250 mg L^−1^ β-naphthol **(1)** in 50 mM potassium phosphate buffer at pH 8. The reaction was incubated for 15 min at 60°C after which the reaction was stopped by heating the solution to 100°C for 5 min to deactivate the enzyme. The resulting precipitate was removed by centrifugation at 13,000 rpm for 10 min.

#### 2.2.1 ^1^H NMR and ^13^C 1D NMR measurements

NMR measurements were done on a 600 MHz Bruker Avance Neo (Billerica, MA, USA) equipped with a cryogenic probe. 1D ^1^H NMR single pulse experiments were performed using 2 s relaxation delay, 1 s acquisition time and eight scans. 1D ^13^C NMR single pulse experiments were performed on the cryoprobe using 2 s relaxation delay, 1 s acquisition time and 1,024 scans. For the processing, an exponential line broadening of 1.0 Hz and no zero-filling was used. All spectra were referenced to TMS, measured at 25°C and processed by MestReNova 12.0 software.

#### 2.2.2 Fourier transform infrared spectroscopy

Fourier transform infrared (FTIR) was utilized in absorbance mode on a Shimadzu IRTracer-100 (Nakagyō-ku, Kyoto, Japan) to verify the successful polymerization of the β-naphthol **(1)** by analyzing the polymer characteristic functional groups (Pham et al., 1993; Premachandran et al., 1996). Measurements were recorded in the range of 500–3,500 cm^−1^ using 64 scans at a resolution of 4 cm^−1^.

#### 2.2.3 Solid-state NMR

The ssNMR measurement was performed on a 600 MHz Bruker Avance Neo spectrometer equipped with a standard bore 14.1 T magnet and a 3.2 mm HCN e-free probe from Bruker Biospin. The corresponding Larmor frequencies were 600.13 and 150.78 MHz for ^1^H and ^13^C, respectively. ^13^C chemical shifts were referenced to TMS externally by calibrating adamantane ^13^C signals.

^1^H–^13^C cross polarization (CP) magic-angle spinning (MAS) ssNMR experiments were done using the following conditions: 4,096 scans, 50 kHz nutation frequency, 1 ms contact time, 3 s repetition delay, 10 ms acquisition time, and 70–100 ramp. In addition, the semi-quantitative experiment was conducted using the MutiCP pulse sequence (Johnson and Schmidt-Rohr, 2014) under the following conditions: 4,096 scans, 62.5 kHz nutation frequency, 7 CP blocks with 1.1 ms each (total CP contact time of 6.6 ms) with 0.7 s z-filter time between CP blocks, 2 s repetition delay, and 10 ms acquisition time. Proton decoupling at 83 kHz was used. The sample was measured at 15 kHz MAS frequency and 277 K. All spectra were processed using Topspin 4.3 using an exponential line broadening of 100 Hz and 32 K zero-filling.

#### 2.2.4 Powder X-ray diffraction

PXRD patterns were acquired using a Rigaku MiniFlex benchtop diffractometer equipped with a Cu *K*α radiation source and a D/teX Ultra2 detector. Samples were packed onto a glass slide with a well size of 20 mm by 20 mm with a 0.5 mm depth. Experiments were conducted with an X-ray voltage of 40 kV and a current of 20 mA, over a range of 3° to 90° 2θ, a step size of 0.010°, and a dwell time of 10 s, which corresponds to an acquisition time of ca. 10 min per sample. The CrystalDiffract software package was used to process the PXRD patterns.

#### 2.2.5 Stepwise optimization of β-naphthol (1) polymerization

The variables tested were temperature, pH and starting enzyme concentration. We started by testing different temperatures in separate experiments at 30, 37, and 60°C. We measured the increase in absorbance at 600 nm over 9 days, taking readings after 4 and 9 days. To test the effect of pH on polymerization, potassium phosphate buffer was adjusted to pH 5, 8, and 10 using an ADWA AD1030 pH meter (Szeged, Hungary) in separate experiments and incubated at a temperature of 37°C and a working enzyme concentration of 440 nM for the same duration as mentioned for the temperature experiments. The final parameter tested was the initial enzyme concentration. We tested three different *Bli-*Lacc concentrations: 50, 440, and 1,000 nM. A temperature of 37°C and a pH of 10 were used as the incubating conditions for different enzyme concentration experiments. Parameters fixed for each variable were changed based on the results of the preceding experiment to ensure optimal conditions for polymer formation. After each set of experiments were completed (9 days), the test tubes were placed in an oven at 90°C until all the buffer was completely evaporated. The colors and properties of the formed residues were assessed.

### 2.3 One gram conversion

A one-gram scale conversion of β-naphthol **(1)** was carried out in a final volume of 500 mL in a screw-cap bottle following previously run procedure with some modifications (Habib et al., 2017). The reaction mixture contained 1 g β-naphthol **(1)** and 1 μM *Bli*-Lacc in 50 mM potassium phosphate buffer adjusted to a final pH of 10. The reaction was incubated at 37°C for 14 days (static state). The reaction mixture was then centrifuged, the supernatant decanted and the precipitate dried in the oven until constant weight to determine the yield.

### 2.4 Crystallization and data collection

Crystallization trials were performed using previously published results on other laccases (Brissos et al., 2022). The sitting-drop method was used with 2 M ammonium sulfate (Sigma Aldrich) as the reservoir and the drop was composed of a 3:1 ratio of protein (5 mg mL^−1^): reservoir solution. All crystallization conditions were conducted at 20°C. Crystals started to appear within a few days and were stable for months in the sitting drop.

The crystals were soaked for ~1 h in a cryoprotectant solution containing 3 M ammonium sulfate and 20% glycerol (v/v). The crystals were directly frozen in the 100°K nitrogen stream. Data was collected using a Bruker D8 VENTURE diffractometer (Billerica, MA, USA) attached to a PHOTON 100 detector. Because of the long unit cell, oscillation images were collected using 0.1° steps, with 6 min exposures, and at a crystal to film distance of 100 mm. The diffraction intensities were integrated and scaled using the associated Bruker software. The crystals had a space group of P6(5) with cell dimensions of a = b = 94.8 Å, c = 271.7 Å, α = β = 90°, γ = 120°.

### 2.5 Structure determination

Using the Basic Local Alignment Search Tool (BLAST) on the NCBI online database, the laccase with the highest sequence similarity to the *Bli-*Lacc was the CotA laccase from *Bacillus subtilis* (PDB: 2X87; Bento et al., 2010). From Matthews probability calculations (Matthews, 1968), it was most likely that there were two copies of the protein in the crystallographic asymmetric unit. Assuming a dimer, two copies of CotA laccase was used for molecular replacement with the PHASER routine (McCoy et al., 2007) within the PHENIX software package (Afonine et al., 2005). This molecular replacement solution was then used as a starting model for refinement. Six amino acid sequences of copper-containing oxidases/laccases were aligned against the *Bli*-Lacc sequence using Clustal Omega (https://www.ebi.ac.uk/jdispatcher/msa/clustalo) and the alignment visualized using Jalview software Version 2.11.4.0 (Waterhouse et al., 2009).

## 3 Results

Polymerization of β-naphthol **(1)** proceeds via free radical formation catalyzed by *Bli*-Lacc. The structure of the polymer gives important insight into the mechanism of its formation as well as helps identify key features needed for possible future applications. In this section, we explain the difference between the distribution of both protons and carbons in the monomer and formed polymer using ^1^H and ^13^C 1D NMR spectroscopy. This is then followed by a detailed FTIR analysis to assess the functional groups found in the polymer. Solid-state NMR was used to explore fine details not seen by conventional NMR analyses. Powder XRD was used to determine the form of the polymer (e.g., amorphous or crystalline).

### 3.1 Elucidation of the structure of poly-β-naphthol (3,4)

**Figure 1:**
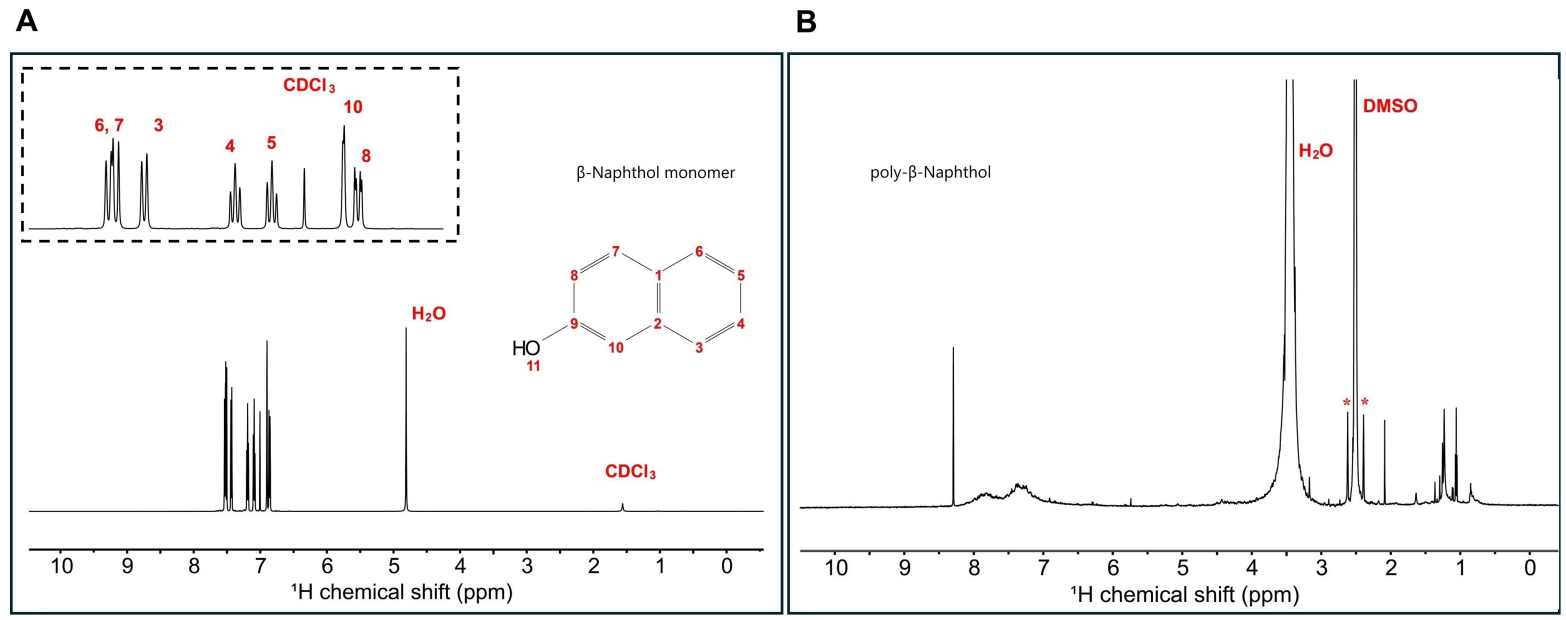
**(A)** ^1^H 1D single pulse NMR experiment for β-naphthol **(1)** dissolved in CDCl_3_ and H_2_O. Inset shows the aromatic region peak assignments as follows: H8 (6.86 ppm, d, 12 Hz, 1), H10 (6.9 ppm, s, 1), H5 (7.09 ppm, t, 6 Hz, 1), H4 (7.19 ppm, t, 6 Hz, 1), H3 (7.43 ppm, d, 12 Hz, 1), H6 (7.51 ppm, d, 12 Hz, 1), and H7 (7.53 ppm, d, 12 Hz, 1). **(B)** ^1^H 1D single pulse NMR experiment for β-naphthol precipitate dissolved in DMSO-*d*_6_. The sample was referenced to DMSO at 2.49 ppm. H_2_O appears at 3.33 ppm and the asterisks correspond to the satellite transitions of DMSO.

**Figure 2:**
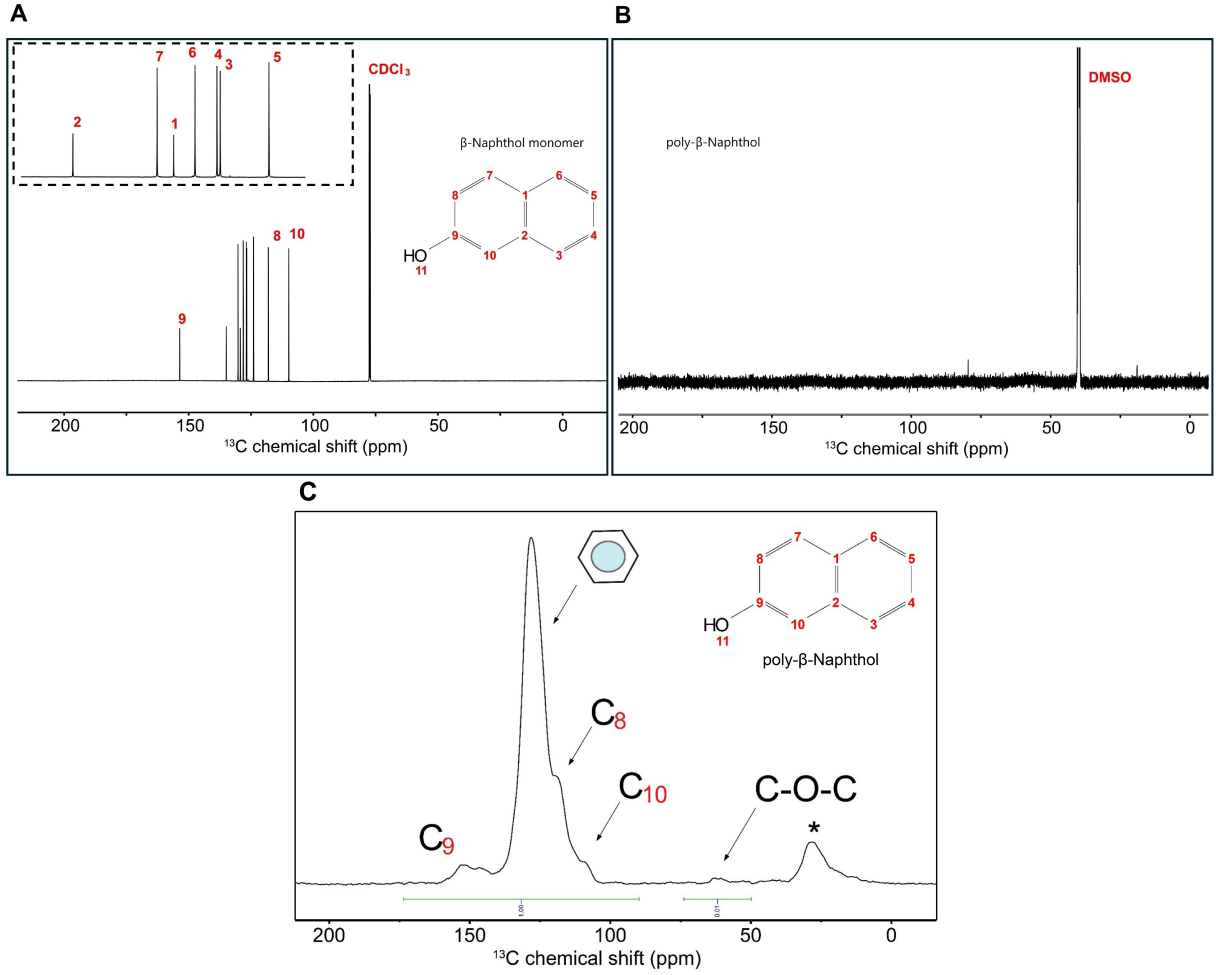
**(A)** ^13^C 1D single pulse NMR experiment for β-naphthol **(1)** dissolved in CDCl_3_ and H_2_O. Inset shows the region from 120 to 140 ppm with the peak assignment as follows: C10 109.9 ppm, C8 118 ppm, C5 124 ppm, C3 126.7 ppm, C4 126.9 ppm, C6 128 ppm, C1 129.3 ppm, C7 130.3 ppm, C2 135 ppm, and C9 153.6 ppm. **(B)** ^13^C 1D single pulse NMR experiment for β-naphthol precipitate dissolved in DMSO-*d*_6_. The sample was referenced to DMSO at 39.5 ppm. **(C)** 1D MultiCP ssNMR spectrum for poly-β-naphthol **(3,4)** powder showing the different functional groups in the polymer including the ether linkages and phenolics. ^*^means spinning sidebands.

β-naphthol **(1)** structure was characterized using ^1^H and ^13^C 1D NMR spectroscopy to assign all the chemical shifts. In Figure 1A, H11 does not appear in the β-naphthol **(1)** sample due to its rapid exchange with the water molecules, while it appears in the β-naphthol precipitate in Figure 1B as the sample was dissolved in DMSO-*d*_6_ (aprotic solvent). Carbons C1, C2, and C9 are quaternary carbons so they possess no protons. Protons H3 (7.43 ppm, d, 12 Hz, 1), H4 (7.19 ppm, t, 6 Hz, 1), H5 (7.09 ppm, t, 6 Hz, 1), H6 (7.51 ppm, d, 12 Hz, 1), H7 (7.53 ppm, d, 12 Hz, 1), H8 (6.86 ppm, d, 12 Hz, 1), and H10 (6.9 ppm, s, 1) are assigned according to the literature (Premachandran et al., 1996). In Figure 2A, the ^13^C peak assignment according to the literature is as the following; C1 129.3 ppm, C2 135 ppm, C3 126.7 ppm, C4 126.9 ppm, C5 124 ppm, C6 128 ppm, C7 130.3 ppm, C8 118 ppm, C9 153.6 ppm, and C10 109.9 ppm (Premachandran et al., 1996).

^1^H spectrum (Figure 1B) for poly-β-naphthol **(3,4)** precipitate dissolved in DMSO-*d*_6_ showed a broad peak in the aromatic region between 6.5 and 8.5 ppm indicating the presence of a polymeric compound formed from β-naphthol **(1)**. A sharp and high intensity peak at 8.29 ppm was assigned to the proton in the hydroxylic functional group (H11). This peak appeared in the aprotic solvent, DMSO-*d*_6_, as it was not exchanging with the solvent surrounding it. In Figure 2B, the ^13^C spectrum showed no peaks in the aromatic region between 120 and 150 ppm. This could indicate the high molecular weight for the formed polymeric material.

**Figure 3:**
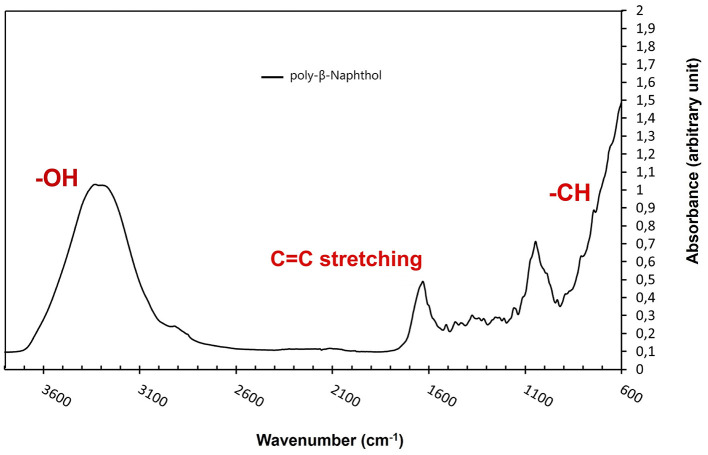
FTIR spectrum of poly-β-naphthol **(3,4)** (black line) produced via laccase mediated enzymatic polymerization.

**Scheme 1:**
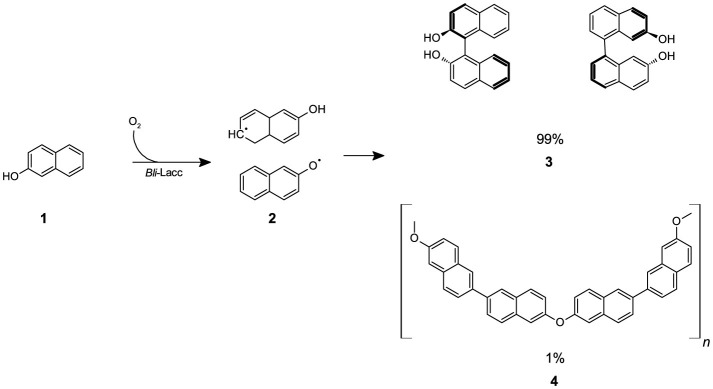
The reaction of β-naphthol **(1)** with CotA-laccase (*Bli-*Lacc) produces free-radicals **(2)** that polymerize to give a polymer product **(3,4)**.

As depicted in Figure 3, the presence of a prominent OH stretching region in the polymer signifies its notable naphthol character (Premachandran et al., 1996). Furthermore, the OH stretching region exhibited a shift to higher frequencies in comparison to the monomer, likely due to weakened hydrogen bonding interactions among the hydroxyl groups within the polymer, as opposed to the self-associations present in the monomer. A notable observation pertains to the bands within the 900–650 cm^−1^ range, attributed to CH out-of-plane bending frequencies. These bands are distinct indicators of substitution patterns in an aromatic ring. The disappearance of the 844 cm^−1^ band, corresponding to the CH out-of-plane bending of a lone hydrogen upon polymerization, suggests the involvement of the 1 position in the polymerization process. The bands at 814 and 748 cm^−1^, however, remain intact. Notably, there is a discernible peak with very low intensity between 1,270 and 1,280 cm^−1^, signifying the presence of low content of aromatic ether linkages (Ar-O-Ar). The polymer still retains vibrational bands indicative of unbound naphthol hydroxyl groups, including the phenolic CO stretch at ~1,210 cm^−1^ and the OH stretch spanning from 3,100 to 3,500 cm^−1^, thus confirming its fundamental naphthol nature. After careful analysis, the peak at around 1,050 cm^−1^ is attributed to C-O stretching in alcohols and was due to ethanol traces from the reaction mixture. Furthermore, the peak at ~ 1,060 cm^−1^ is attributed to the C=C stretching in aromatic compounds. Based on NMR and FTIR results, we depicted a hypothetical structure for our generated poly-β-naphthol **(3,4)** (Scheme 1).

#### 3.1.1 Solid-state NMR and XRD analyses

SsNMR experiment showed the different functional groups in the polymer including ether linkages at 63 ppm and phenolics/oxyaromatics at 146 and 153 ppm (Figure 2C). The integration of ether linkages to the aromatic region of poly-β-naphthol **(3,4)** corresponded to around 1% of the total area. Two peaks appeared in the phenolics region (145–165 ppm) at 147 and 151 ppm in poly-β-naphthol **(3,4)**, respectively, while only a single peak appeared in the carbon spectrum of β-naphthol **(1)** in solution NMR spectroscopy. This indicated that the hydroxyl functional groups have different orientations in the same polymer. The comparison between CP and MultiCP spectra showed that MultiCP gave an enhanced sensitivity and resolution over the CP with more resolved peaks in the aromatic region (105–145 ppm) where different carbon peaks could be identified and assigned at 109 and 119 ppm corresponding for ortho-oxyaromatics C_10_ and C_8_, respectively (Supplementary Figure 2). Powder X-ray diffractogram for poly-β-naphthol **(3,4)** showed that the sample was mainly amorphous except for a small portion showing diffraction peaks (Supplementary Figure 3).

### 3.2 Optimization of poly-β-naphthol (3,4) formation

To maximize yield and attain the highest degree of polymerization of β-naphthol **(1)**, optimization experiments using various parameters must be conducted. To do this, three levels of each parameter were selected covering high, middle and low ends. The variables chosen were temperature, pH and enzyme concentration. Polymer yield was determined based on the OD_600nm_ readings after 4 and 9 days. Out of the different variables tested, the optimal conditions for polymerization of β-naphthol **(1)** were found to be a temperature of 37°C, a working pH of 10, and an enzyme concentration of 440 nM over a period of 9 days (see Supplementary Table 1). Different colors of residue formed after drying from the different reaction conditions were recorded (Supplementary Figure 4). Further future analysis of these residues by NMR could reveal different sized polymers.

### 3.3 One gram conversion

One-gram conversions are used to determine if a reaction could be extrapolated to a pilot scale for industrial applications. To apply this hypothesis, a one-gram scale conversion of the β-naphthol **(1)** polymerization was conducted. It was found that one gram of β-naphthol **(1)** produced 216 mg poly-β-naphthol **(3,4)** (≈ 21.6% conversion yield) as dry weight.

To be able to study and elucidate important characteristics/features of the *Bli*-Lacc enzyme, a crystal structure needs to be solved. To achieve this, the purity level of the expressed enzyme needs to be enhanced. This is achieved using IMAC followed by ion-exchange chromatography to remove any non-specifically bound proteins from the column. The purified enzyme is then crystallized followed by X-ray diffraction. Analysis of the structure using computational tools can then identify molecular details important for determining function, binding and other parameters necessary for laccase activity.

### 3.4 Purification of *Bli*-Lacc for crystallization

**Scheme 2:**
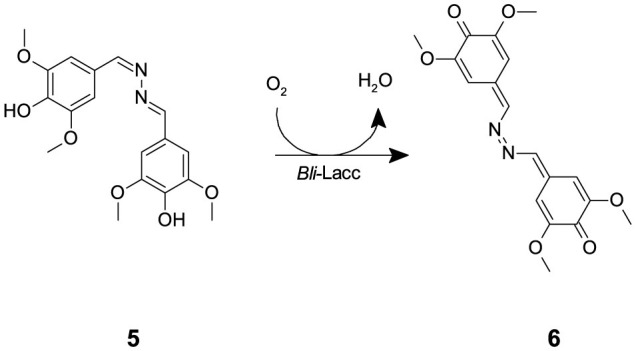
The laccase mediated conversion of SGZ **(5)** into TMAMQ **(6)**.

The expressed enzyme was pure after running through the monoQ anion exchange column, evident through crystal formation within 2–3 days using the sitting drop technique. Purity of the protein fractions eluted from the monoQ purification was assessed upon measuring activity using syringaldazine (SGZ) **(5)** as a substrate. SGZ **(5)** is known for its ability to detect laccase and peroxidase activities (Harkin et al., 1974; Leonowicz and Grzywnowicz, 1981). Laccase oxidation of SGZ **(5)** is detected by the formation of a violet color. This violet color corresponds to the formation of tetramethoxy azobismethylene quinone (TMAMQ) **(6)** (Scheme 2) a compound known for its ability to measure antioxidant activity in various foods (Nugroho Prasetyo et al., 2010). Using SGZ **(5)** as an indicator for presence of laccase activity, each of the fractions that were run on the protein gel were assessed for their ability to convert SGZ **(5)** to TMAMQ **(6)** based on violet color formation/intensity. All fractions collected before fraction 32 showed no laccase activity but starting from fraction 34 onwards, all fractions showed positive activity (Supplementary Figure 5). However, based on the protein gel, we decided to pool only fractions 40–42 having the highest and purest yield of intact laccase. The pooled fractions 40–42 were dialyzed against Tris (20 mM) pH 7.5, NaCl (200 mM) buffer. The protein was concentrated using centricon filter tubes to a final concentration of 5 mg mL^−1^ (OD 1). The blue color of the protein was evident in this step indicating the successful binding of the copper to the protein.

### 3.5 Atomic structure

**Figure 4:**
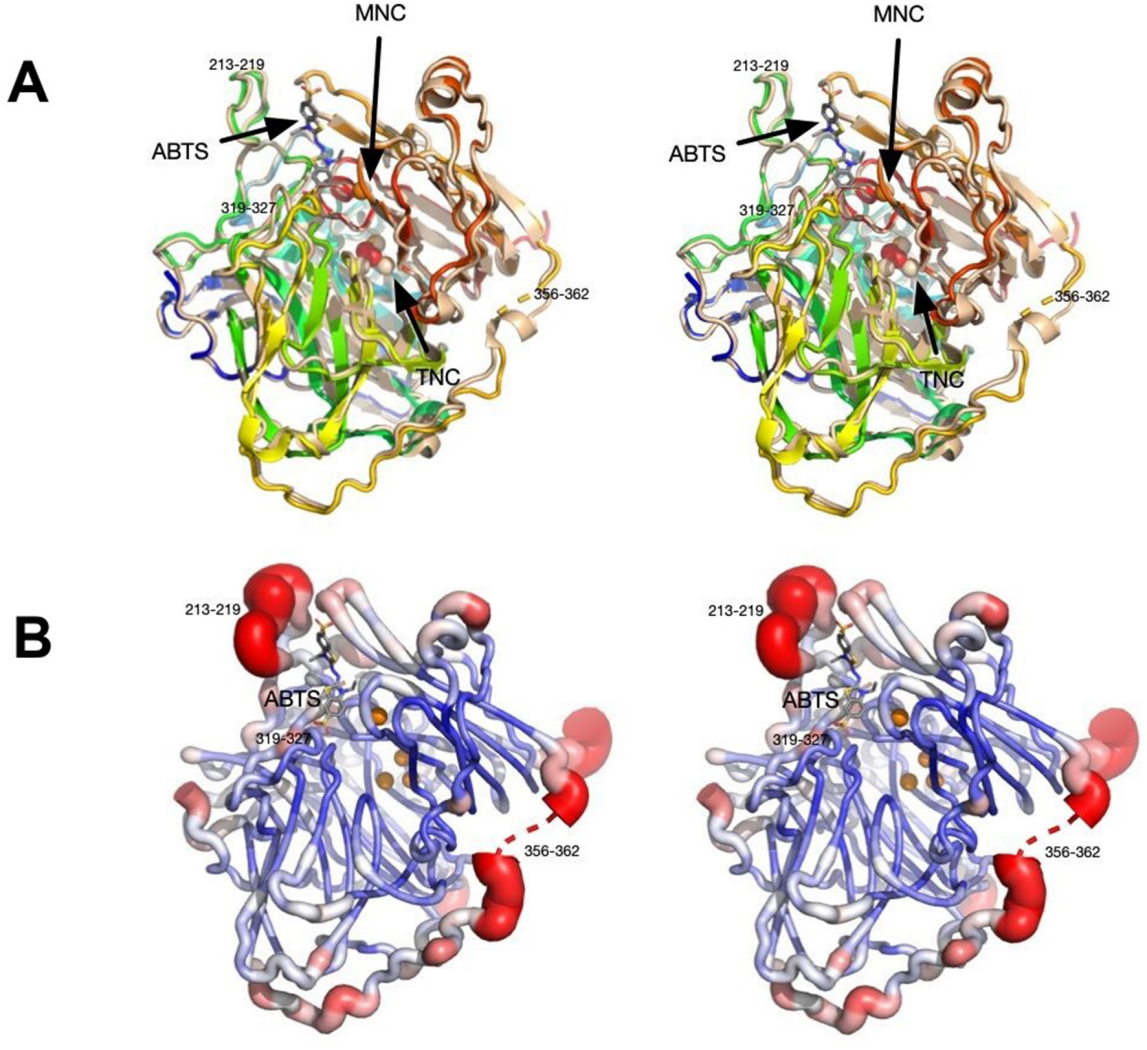
Stereo images showing structural features of *Bli*-Lacc. **(A)** 3D alignment of *Bli*-Lacc with CotA laccase from *Bacillus subtilis* (PDBID:3ZDW) that was complexed with ABTS. Both structures are represented as ribbon diagrams with *Bli*-Lacc colored from blue to red as the chain extends from the N to C termini. The *B. subtilis* structure is shown in wheat color with its bound ABTS represented by stick figures and the bound oxygen as red spheres. The two copper redox centers (MNC and TNC) are labeled. Noted are the loops significantly different to each other; 356–362, 213–219, and 319–327. **(B)** Areas of mobility in the *Bli*-Lacc structure as per the refined B values for each residue. The most mobile regions are colored red and represented by thicker tubes while the areas with the smallest B values are dark blue, thin tubes.

Using the structure of CotA laccase from *B. subtilis* (PDB ID: 2X87; Bento et al., 2010), the PHASER routine within PHENIX found the predicted dimer in the P6(5) asymmetric unit. The data and refinement statistics are summarized in Supplementary Table 2. The current model at 2.7 Å resolution has an Rmodel of ~17% and an Rfree of ~23%. The model consisted of residues 2–509 and therefore the residues beyond 513 that were added during cloning were not visible in the density. As found in several other laccase structures, two loops were disordered hence not interpreted with an atomic model: residues 90–96 and 359–361. Not unexpectedly, the structure of *Bli*-Lacc was highly similar in sequence to other laccase structures. Shown in Figure 4A is a structure alignment of *Bli*-Lacc with CotA laccase from *B. subtilis* (PDB ID: 3ZDW) that was complexed with 2,2′-azinobis-(3-ethylbenzothiazoline-6-sulfonate; ABTS; Enguita et al., 2003). The root mean square deviation (RMSD) between the two structures was 0.5 Å sharing 66% sequence identity and 78% similarity. While the structures of *Bli*-Lacc complexed with other substrates was also attempted, the substrates were insoluble in the high concentration of ammonium sulfate used for crystallization. Noted in this figure are the locations of the mononuclear (MNC) and trinuclear (TNC) redox centers. Also noted are the areas with the largest divergence between the two structures. Notably, two loops, 213–219 and 319–327, are immediately adjacent to the substrate binding site. These are unlikely due to substrate binding since those loops in *B. subtilis* are the same in the presence and absence of substrate. This suggests possible differences in substrate specificity and/or affinity.

With this sequence variability and structural differences in the substrate binding pocket compared to *B. subtilis* CotA, the apparent structural flexibility was examined. Shown in Figure 4B, is a representation of conformational flexibility as per the refined B factors in the structure. Here the most flexible (i.e., highest B values) regions are colored red and represented by “swollen” tubes. Aside from the disordered region at 356–362, the most flexible is the 213–218 loop adjacent to the substrate binding pocket. Therefore, the most divergent and conformationally flexible regions appear to be involved with substrate selectivity and affinity.

**Figure 5:**
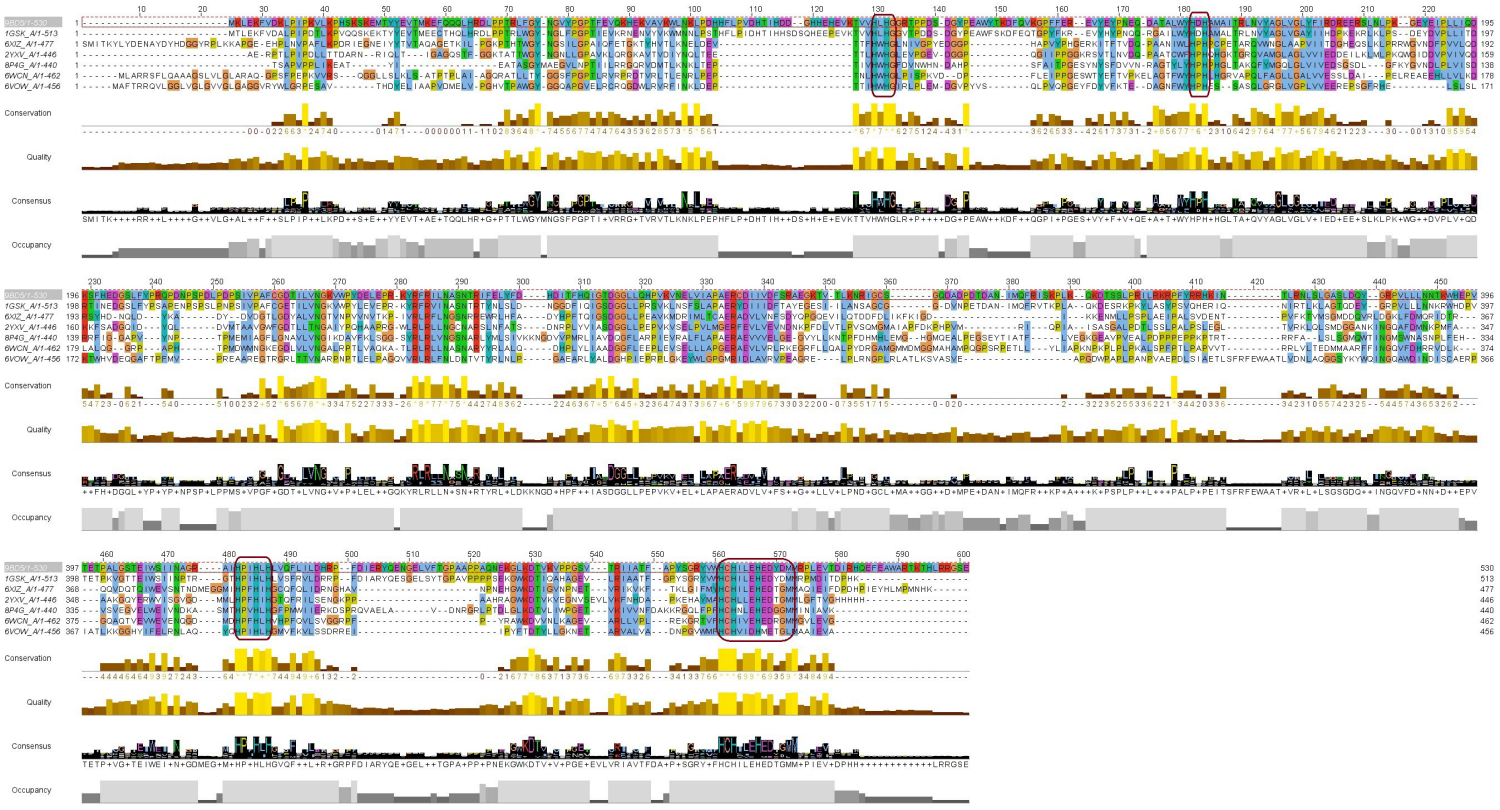
A multiple sequence alignment of the *Bli*-Lacc amino acid sequence against six copper containing oxidases/laccases of solved structures deposited in the PDB database. Boxes (magenta outline) show the motifs: HXHG, HXH, HXXHXH, and HCHXXXHXXXXM/L/F, that form the four copper ligands and are highly conserved in laccases.

To further examine the overall sequence similarity amongst the various sources of laccase, the structure was input into the CONSURF Server (https://consurf.tau.ac.il/consurf_index.php; Landau et al., 2005; Ashkenazy et al., 2010, 2016; Celniker et al., 2013; Yariv et al., 2023). For comparison, 150 sequences were aligned to *Bli*-Lacc (Supplementary Figure 6A) and the similarity between the sequences projected onto a ribbon diagram (Supplementary Figure 6B). For each residue in *Bli*-Lacc, the highest percent of a particular amino acid was calculated. The most highly conserved residues are in red, and the least are in cyan. As expected, the most highly conserved residues are those in the vicinity of the bound metals and/or those in the core of the protein. To further validate and confirm the resemblance of the *Bli*-Lacc against other laccases with solved crystal structures, a multiple sequence alignment using the amino acid sequences was executed and motifs characteristic of laccases were found conserved—HXHG, HXH, HXXHXH, and HCHXXXHXXXXM/L/F as can be seen in Figure 5 (Sharma et al., 2007).

## 4 Discussion

Polymerization of aromatic and aliphatic compounds to produce products of industrial importance represents a challenge as well as an opportunity in the biotechnology field (Nicell et al., 1993). Bicyclic aromatic compounds such as α- and β-naphthol **(1)** present a menace as environmental pollutants (Qi et al., 2023). Polymerization of these compounds is one way for their elimination and transformation to more valuable products. Biocatalytic polymerizations have gained increasing attention in recent years (Nikulin and Švedas, 2021). The ability to produce compounds with high purity and enantioselectivity is crucial for many industries (Kobayashi et al., 2001). Owing to the green nature of enzymes, we attempted the polymerization of β-naphthol **(1)** using a single oxidative enzyme, namely a CotA-laccase from *Bacillus licheniformis* ATCC 9945a.

In a single step, we successfully produced a polymer that we fully characterized using ^1^H NMR, ^13^C NMR, ssNMR, PXRD, and FTIR spectroscopy and confirmed the production of poly-β-naphthol **(3,4)**. FTIR data aligns with previously reported IR data concerning the electrochemical oxidation of β-naphthol **(1)**, which also propose the plausible formation of quinonoid moieties (Pham et al., 1993). ssNMR revealed fine details in the form of ether linkages and various orientations for the hydroxyl groups in the overall structure. ssNMR provided insights into the polymer structure, suggesting that ~98% of the polymer consisted of polyhydroxyphenylene, indicating that polymerization occurred predominantly on the carbons of the aromatic ring. Small amounts of ether linkages were also observed in the polymer. These findings are consistent with the solubility characteristics of the polymer. Our polyhydroxyphenylenes were insoluble in tetrahydrofuran (THF) and chloroform but were soluble in dimethyl sulfoxide (DMSO; Kovacic and Kyriakis, 1962, 1963), whereas polyphenylene ethers are known to be soluble in THF (Hay et al., 1959). Furthermore, the XRD pattern shown in Supplementary Figure 3 indicates that the polymer was primarily amorphous. The XRD pattern also revealed a d-spacing in the range of 4.4–4.5 Å, which corresponds to the length of the phenyl ring consistent with previously reported studies (Marvel and Hartzell, 1959; Kovacic et al., 1968). Optimization by modifying conditions of temperature, pH and enzyme concentration was conducted to improve the yield of polymer formation. The variables were chosen based on high, medium and low-end levels to determine the optimal conditions for polymerization of β-naphthol **(1)**. The one-gram conversion was performed over 14 days based on our initial run before further optimization was done. However, based on the results generated in our 14-day experiments, a constant level of absorbance at 600 nm can be seen starting from day 9. As a result, the data generated for the one-gram conversion over 14 days can be considered more or less similar to that obtained after 9 days. The initial experiment runs done before further optimization can be seen in Supplementary Figure 7. Formerly, the mechanical properties of lignin-asphalt mixtures were studied and contradictory results were seen (Zhang et al., 2020). The incorporation of poly-β-naphthol **(3,4)** may thus have better properties when combined with asphalt but this entails a future, more comprehensive study. Nonetheless, working under different conditions yielded different colored products indicative of polymers with different molar masses hence possibly different properties (Jones and Kovacic, 1989).

In our conversion, the yield could be seen as relatively low as compared to other chemical or possibly physical approaches of polymeric synthesis (Qi et al., 2023). However, this biological method of production of poly-β-naphthol **(3,4)** not only is green but rather economic. The ability to transform simple compounds/substrates into polymers with industrial applications is currently a hot topic in both academia and industry (Dijkman et al., 2014; Ricklefs et al., 2015, 2016; Habib et al., 2017, 2018; Guo et al., 2023; Marić et al., 2023; Petermeier et al., 2024).

Previously, CotA-laccases have been characterized and several structures have been solved for this class of enzymes (Martins et al., 2002; Enguita et al., 2003; Liu et al., 2016). Although the expression level of this enzyme is not relatively high, it can still perform biocatalytic conversions at a fairly good level. It also managed to form crystals in a relatively short period of time using former protocols used for laccase crystallization (Brissos et al., 2022). Comparative analysis with other laccases using the CONSURF server and multiple sequence alignment, displayed a major degree of resemblance between our expressed laccase and former enzymes of this class with solved crystal structures. The motifs characteristic for copper binding in laccases are present in *Bli*-Lacc and validate our findings in the protein structure (Sharma et al., 2007). This work contributes to the growing body of research on green chemistry, emphasizing the importance of developing environmentally friendly and efficient methods for polymer production. Future studies should continue to explore and optimize these processes, paving the way for broader applications and improved industrial practices.

## 5 Conclusion

The polymerization of β-naphthol **(1)** to its polymeric form **(3,4)** represents a beneficial application for the use of recombinant laccases in polymerization reactions of complex organic compounds. Currently, there is a trend to direct most synthetic reactions and conversions to sustainable and cost-efficient routes. The reactions they perform are selective and the turnover number is quite high enabling a single reaction to be repeated multiple times using the same batch of enzyme. Enzyme immobilization has facilitated recycling of enzymes and could serve as a potential technique for upscaling this polymerization while conserving enzyme costs. Future work will involve exploring several types of substituted and unsubstituted phenols to understand the mechanism of enzymatic polymerization and monomer structure/activity relationship. Solving the crystal structure of *Bli*-Lacc also adds to the list of recombinant laccases with crystal structures and facilitates understanding of how this enzyme uses its mechanistic machinery to biocatalyze various catalytic conversions.

## Data Availability

The datasets presented in this study can be found in online repositories. The names of the repository/repositories and accession number(s) can be found in the article/Supplementary material.
