# Supplementary material for: Polymerization potential of a bacterial CotA-laccase for β-naphthol: enzyme structure and comprehensive polymer characterization
Source: Front Microbiol. 2024 Nov 21;15:1501112. doi: 10.3389/fmicb.2024.1501112 (PMC11619140; doi:10.3389/fmicb.2024.1501112)
Supplement: Supplementary file 1 [file Supplementary_file_1.docx]

Supplementary Material

Table of Contents

| **Supplementary Figure S1** SDS PAGE of *Bli-*Lacc purified via nickel affinity chromatography | 2 |
| --- | --- |
| **Supplementary Figure S2** CP/MAS and MultiCP 1D ssNMR experiments for poly-β-naphthol **(3,4)** | 3 |
| **Supplementary Figure S3** Powder X-ray diffractogram for poly-β-naphthol **(3,4)** | 4 |
| **Supplementary Figure S4** Residues formed from the reaction of *Bli*-Lacc with β-naphthol **(1)** | 5 |
| **Supplementary Figure S5** Analysis of the different fractions from the monoQ anion exchange chromatography purification of the *Bli*-Lacc enzyme. | 6 |
| **Supplementary Figure S6** Conservation of laccase sequence using *Bli*-Lacc as a search model as calculated using the program CONSURF | 7 |
| **Supplementary Figure S7** Initial experiments for β-naphthol polymer production using β-naphthol and *Bli*-Lacc as starting materials | 8 |
| **Supplementary Table S1** The difference in optical density during the polymerization reaction at various conditions of temperature, pH and enzyme concentration | 9 |
| **Supplementary Table S2** Data and refinement characteristics for *Bli-*Lacc | 10 |


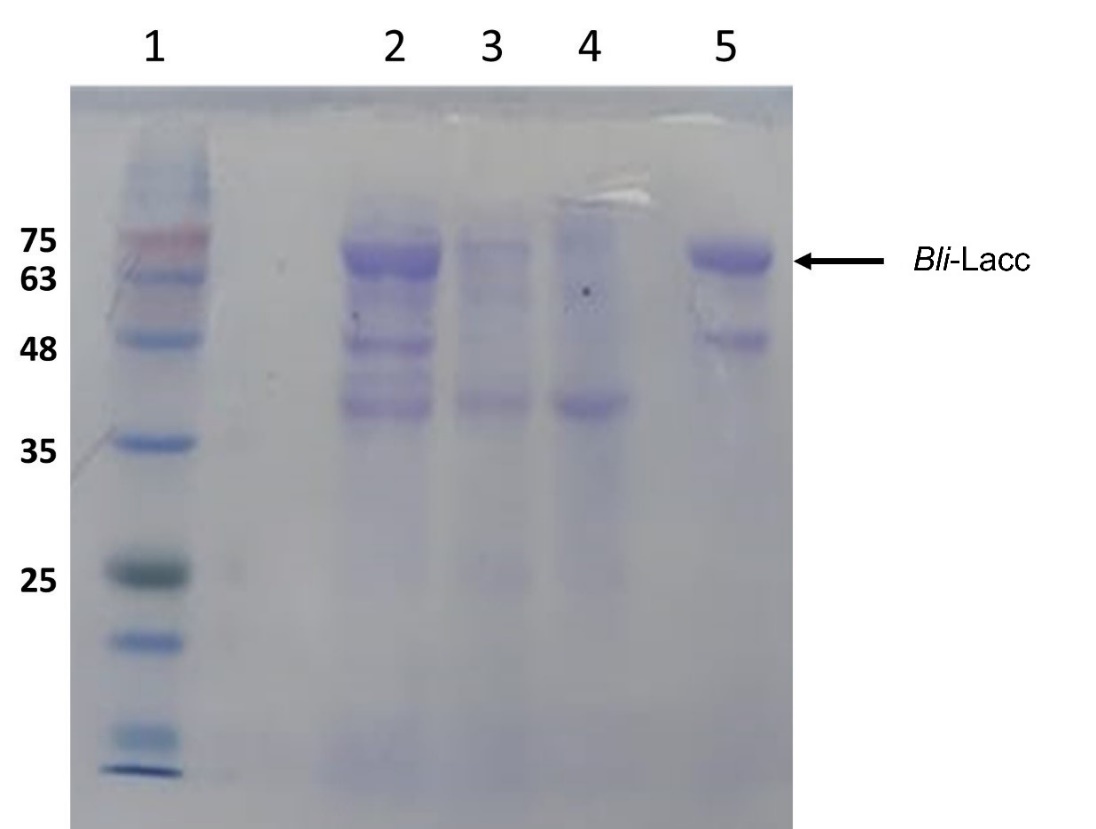


**Supplementary Figure S1.** SDS-PAGE of the various fractions from the purification of *Bli*-Lacc (labelled with an arrow). Legend – lane 1) GoldBio BlueStain^TM^ Protein Ladder, 11-245 kDa (St Louis, MO, USA), lane 2) cell-free extract, lane 3) flowthrough, lane 4) wash, lane 5) eluted protein (nickel affinity column purification)


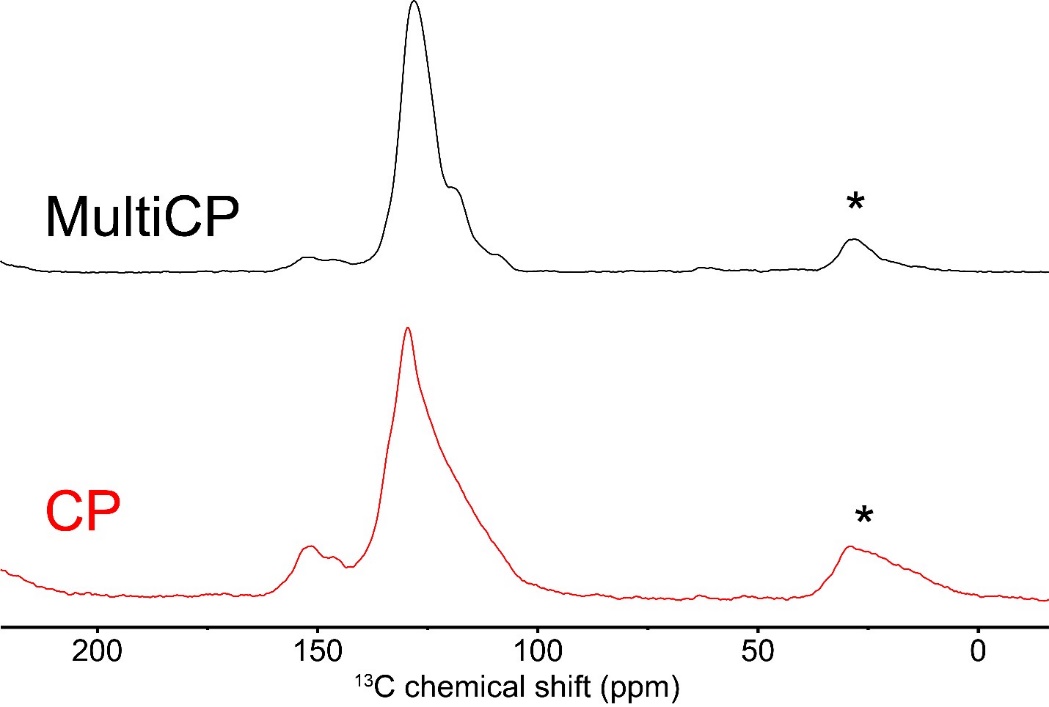


**Supplementary Figure S2** CP/MAS and MultiCP 1D ssNMR experiments for poly-β-naphthol **(3,4)**.

**
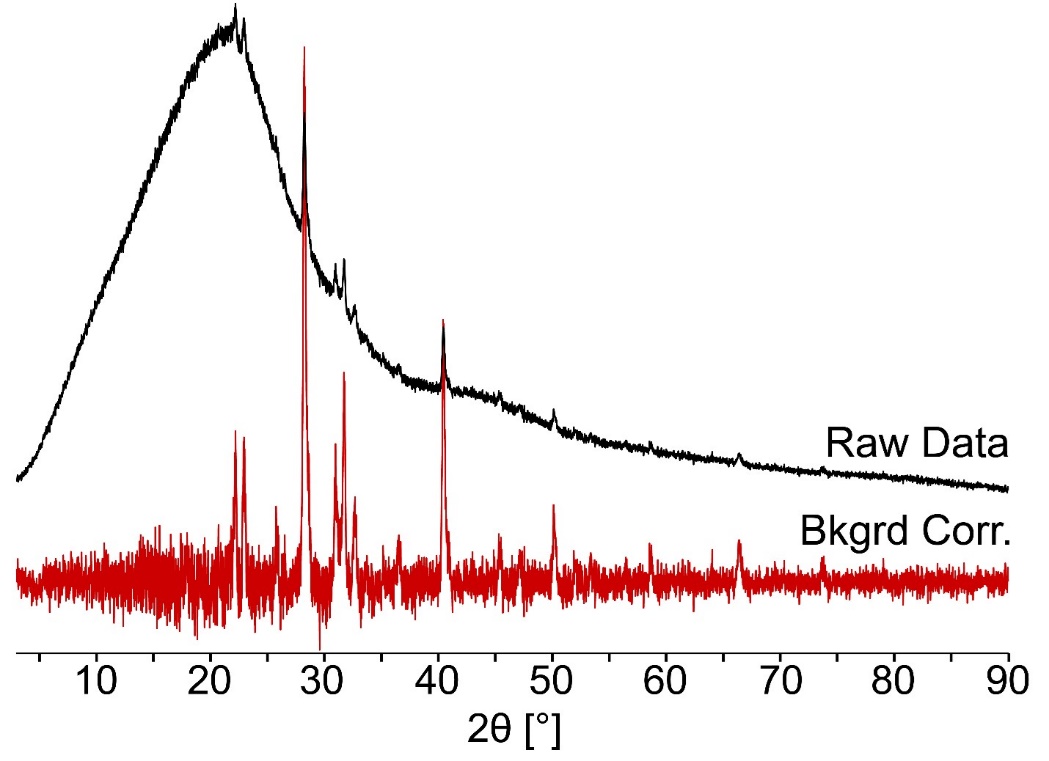
**

**Supplementary Figure S3** Powder X-ray diffractogram for poly-β-naphthol **(3,4)**. The sample is mainly amorphous, but a small portion shows diffraction peaks. The stack plot is for the comparison of the raw data with and without background correction


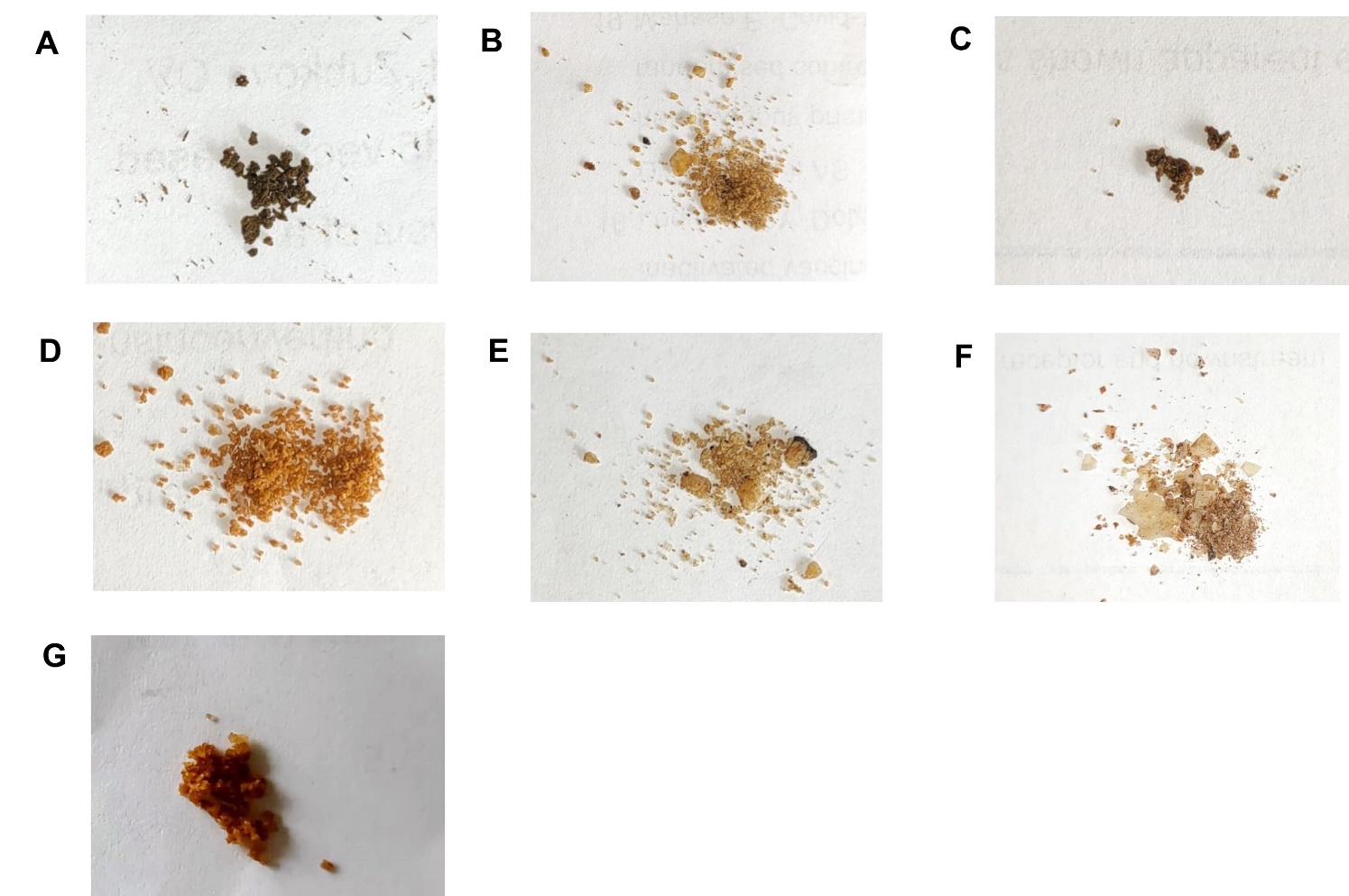


**Supplementary Figure S4** Residues formed from the reaction of *Bli*-Lacc with β-naphthol **(1)** under different working conditions. Conditions are labelled based on Supplementary Table S1.

**
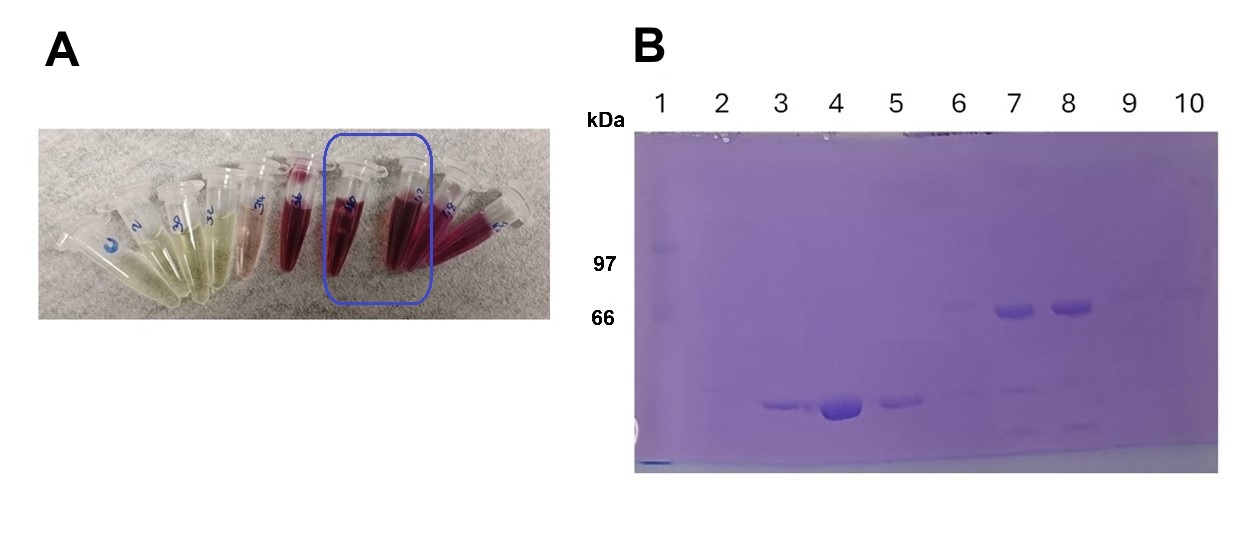
Supplementary Figure S5** Analysis of the different fractions from the monoQ anion exchange chromatography purification of the *Bli*-Lacc enzyme. (A) Syringaldazine assay results showing a positive reaction (yellow to dark pink) for different eluants from the anion exchange (monoQ) column fractions (B) Protein SDS-PAGE gel for different monoQ fractions. Legend: 1 – Prestained SDS-PAGE Standards, low range (Bio-Rad, Hercules, CA, USA), 2 – Frac 2, 3 – Frac 30, 4 – Frac 32, 5 – Frac 34, 6 – Frac 36, 7 – Frac 40, 8 – Frac 42, 9 – Frac 48, 10 – Frac 50. Fractions in lanes 7 and 8 (with tubes circled with a blue line) show the intact enzyme (Mol weight 60 kDa)


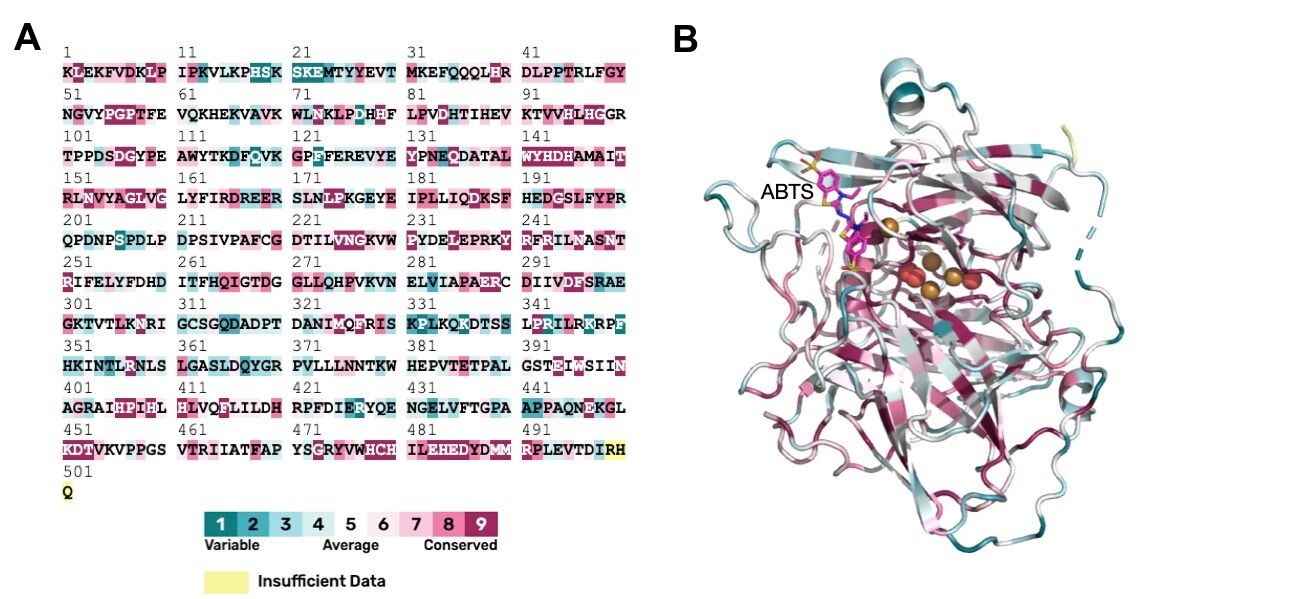
**Supplementary Figure S6** Conservation of laccase sequence using *Bli*-Lacc as a search model as calculated using the program CONSURF. (A) shown here are the aligned sequences using the standard cutoff values. Of the 16,810 homologs collected, 2,733 passed the thresholds while 2,666 are unique hits. For the calculations, 150 hits were used. The average pairwise distance was 1.2Å (0.05-1.8Å). The most highly conserved residues are red, and the least are cyan. (B) shown here is the structure of *Bli*-Lacc color coded as per the results from CONSURF. Overlaid onto the *Bli*-Lacc structure are the bound oxygen (red spheres) and copper (orange spheres) from the 1OF0 structure. Also shown is the model for the bound substrate ABTS


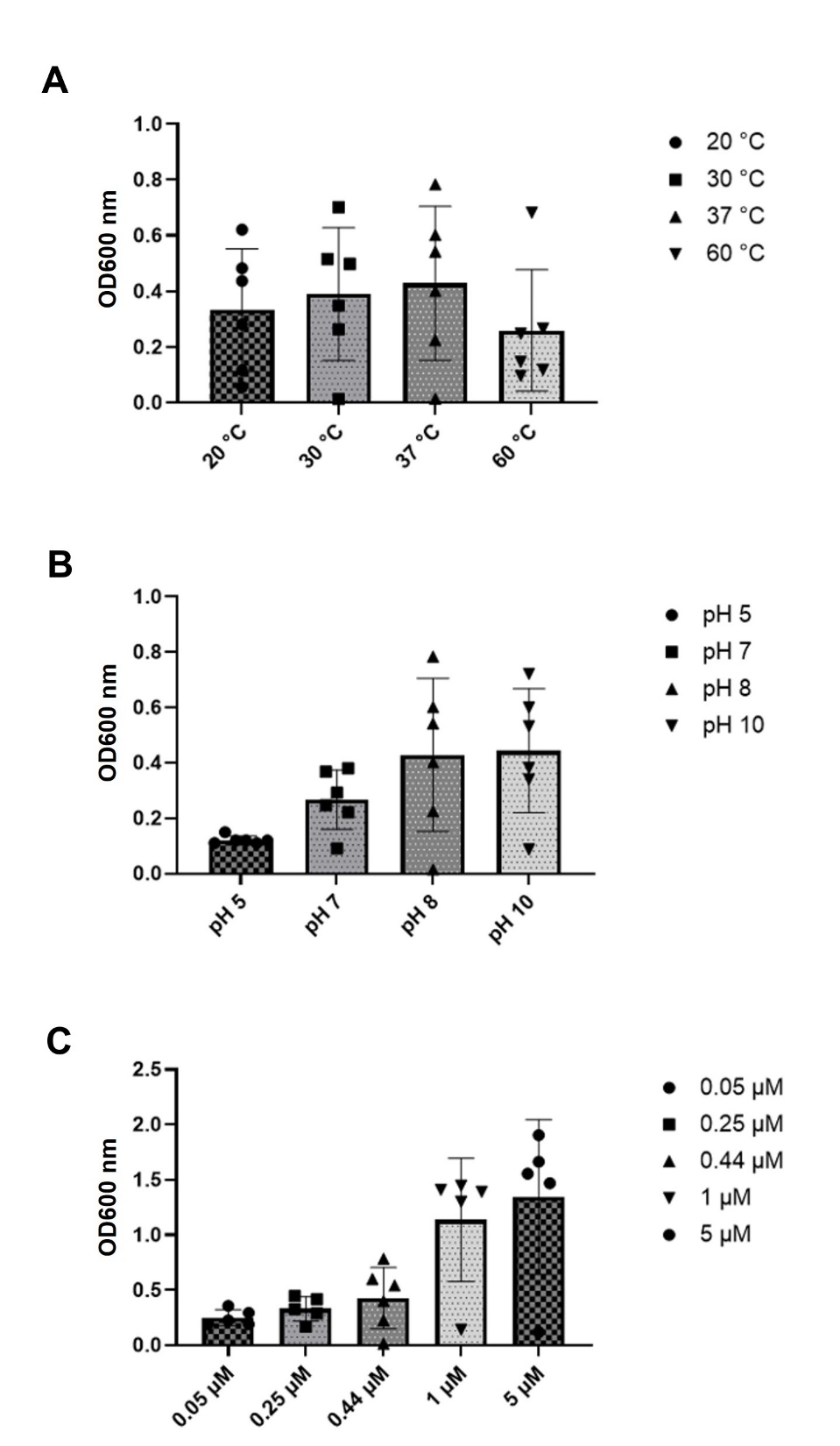


**Supplementary Figure S7** Initial experiments for β-naphthol polymer production using β-naphthol and *Bli*-Lacc as starting materials (A) Effect of temperature on increase in absorbance at 600 nm at 20, 30, 37 and 60 °C (B) Effect of working pH on increase in absorbance at 600 nm at pH 5, 7, 8 and 10 (C) Effect of enzyme concentration on increase in absorbance at 600 nm at 0.05, 0.25, 0.44, 1 and 5 µM concentrations. Results shown represent the average readings over 14 days for each parameter tested

**Supplementary Table S1.** The difference in optical density measured at 600 nm spectrophotometrically during the polymerization reaction at various conditions of temperature, pH and enzyme concentration

| Label | pH | Temperature (°C) | Enzyme Concentration (nM) | OD600 nm  (4 days) | OD600 nm  (9 days) |
| --- | --- | --- | --- | --- | --- |
| (A) | 8 | 30 | 440 | 0.891 | 1.200 |
| (B) | 8 | 37 | 440 | 1.081 | 1.305 |
| (C) | 8 | 60 | 440 | 1.012 | 1.059 |
| (D) | 5 | 37 | 440 | 1.080 | 1.165 |
| (E) | 10 | 37 | 440 | 1.610 | 1.682 |
| (F) | 10 | 37 | 50 | 0.030 | 0.033 |
| (G) | 10 | 37 | 1000 | 0.596 | 0.616 |

**Supplementary Table S2.** Data and refinement statistics for *Bli-Lacc*

|  | PDB ID: 9BD5 |
| --- | --- |
| Wavelength | 1.5406Å |
| Resolution range | 23.7- 2.7 (2.77- 2.7) |
| Space group | P 65 |
| Unit cell | 94.8, 94.8, 271.7, 90, 90, 120 |
| Total reflections | 266407 |
| Unique reflections | 36830 (2752) |
| Multiplicity | 3.8 |
| Completeness (%) | 97.67 (95.89) |
| Mean I/sigma(I) | 9.1 (1.7) |
| Wilson B-factor | 22.45 |
| R-merge | 11.4% (35%) |
| Reflections used in refinement | 36830 (2752) |
| Reflections used for R-free | 1846 (140) |
| R-work | 0.1687 (0.2185) |
| R-free | 0.2300 (0.2933) |
| Number of non-H atoms | 8858 |
| macromolecules | 8180 |
| ligands | 123 |
| solvent | 555 |
| Protein residues | 1002 |
| RMS(bonds) | 0.008 |
| RMS(angles) | 0.97 |
| Ramachandran favored (%) | 93.03 |
| Ramachandran allowed (%) | 5.76 |
| Ramachandran outliers (%) | 1.21 |
| Rotamer outliers (%) | 1.00 |
| Clashscore | 7.22 |
| Average B-factor | 23.03 |
| macromolecules | 22.47 |
| ligands | 49.99 |
| solvent | 25.31 |
